# Molecular evolution in *Panagrolaimus *nematodes: origins of parthenogenesis, hermaphroditism and the Antarctic species *P. davidi*

**DOI:** 10.1186/1471-2148-9-15

**Published:** 2009-01-16

**Authors:** Samantha C Lewis, Leslie A Dyal, Caroline F Hilburn, Stephanie Weitz, Wei-Siang Liau, Craig W LaMunyon, Dee R Denver

**Affiliations:** 1 Department of Zoology and Center for Genome Research and Biocomputing, Oregon State University, Corvallis, Oregon, 97331, USA; 2 Department of Biological Sciences, California State Polytechnic University, Pomona, California, 91768, USA

## Abstract

**Background:**

As exemplified by the famously successful model organism *Caenorhabditis elegans*, nematodes offer outstanding animal systems for investigating diverse biological phenomena due to their small genome sizes, short generation times and ease of laboratory maintenance. Nematodes in the genus *Panagrolaimus *have served in comparative development and anhydrobiosis studies, and the Antarctic species *P. davidi *offers a powerful paradigm for understanding the biological mechanisms of extreme cold tolerance. *Panagrolaimus *nematodes are also unique in that examples of gonochoristic, hermaphroditic and parthenogenetic reproductive modes have been reported for members of this genus. The evolutionary origins of these varying reproductive modes and the Antarctic species *P. davidi*, however, remain enigmatic.

**Results:**

We collected nuclear ribosomal RNA gene and mitochondrial protein-coding gene sequences from diverse *Panagrolaimus *species and strains, including newly discovered isolates from Oregon, to investigate phylogenetic relationships in this nematode genus. Nuclear phylogenies showed that the species and strains historically identified as members of *Panagrolaimus *constitute a paraphyletic group, suggesting that taxonomic revision is required for *Panagrolaimus *and related nematode lineages. Strain-specific reproductive modes were mapped onto the molecular phylogeny to show a single origin of parthenogenesis from a presumably gonochoristic ancestor. The hermaphroditic strains were all placed outside a major monophyletic clade that contained the majority of other *Panagrolaimus *nematodes. Phylogenetic analyses of mitochondrial sequences showed that substantial molecular and geographic diversity exists within the clade of parthenogenetic strains. The Antarctic species *P. davidi *was found to be very closely related to two *Panagrolaimus *strains from southern California. Phylogenetic and molecular clock analyses suggested that *P. davidi *and the California strain PS1579 shared a common ancestor in the very recent evolutionary past.

**Conclusion:**

Our study provides a phylogenetic framework for understanding the evolutionary origins and diversification patterns of varying reproductive modes within *Panagrolaimus *and important insights into the origin of the Antarctic species *P. davidi*. *Panagrolaimus *offers a powerful nematode model for understanding diverse evolutionary phenomena including the evolution of asexuality and the adaptive evolution of extreme cold tolerance.

## Background

The nematode *Caenorhabditis elegans *has long served as an exceptional model system for understanding diverse biological phenomena such as development, neurobiology and genome structure [1-3]. Over the last decade nematodes in the *Caenorhabditis *genus have come to serve as increasingly powerful evolutionary models for studying diverse processes ranging from the mutation rate [4-6] to the evolution of reproductive mode transitions [7,8] to speciation mechanisms [9-11]. Although *C. elegans *and *C. briggsae *were originally thought to share a common origin of hermaphroditic reproduction, more recent molecular phylogenetic approaches have shown independent origins of hermaphroditism for these two *Caenorhabditis *species [12]. There exists over one million genetically diverse nematode species outside of *Caenorhabditis *[13,14] that are also coming to serve roles as models for other evolutionary phenomena. For example, nematodes in the genera *Strongyloides *and *Rhabditophanes *are growing models for understanding the evolution of parasitism [15]; *Oscheius *and *Pristionchus *nematodes offer strong models for investigating the evolution of developmental processes [16-19]. Multiple independent evolutionary transitions from gonochoristic to hermaphroditic reproduction have also been observed in the latter two nematode genera [17,20] and a broad review of numerous rhabditid nematodes showed up to ten independent transitions to hermaphroditism [21].

*Panagrolaimus *nematodes have also played roles in comparative evolution of development studies [16,22,23], and *Panagrolaimus *constitutes the only nematode genus in which three different reproductive modes (gonochoristic, hermaphroditic and parthenogenetic) have been reported. Well-studied parthenogenetic strains of *Panagrolaimus *include a number of isolates from North America (e.g. PS1579) as well as *P. davidi *from Antarctica; the latter has been shown to reproduce through meiotic parthenogenesis [24]. More recently this group has emerged as a model for understanding the physiological and molecular mechanisms underlying anhydrobiosis and extreme cold tolerance, due in part to the discovery of *P. davidi *in Antarctica. This species has evolved multiple strategies to deal with extreme cold including cryoprotective dehydration and the prevention and tolerance of intracellular freezing in adults and eggs [25-33]. A recent comparative analysis of extreme cold tolerance showed that the majority of individuals in experimental lab populations of *P. davidi *survived -15°C treatments (~80% survival) whereas much lower survival rates (all < 20%) were observed in five other nematode species surveyed, including *P. davidi*'s congener *P. rigidus *[25]. *Panagrolaimus *strain PS443 survived over eight years in a state of anhydrobiosis [34].

A previous phylogenetic analysis of *Panagrolaimus *species and strain relationships based on ribosomal RNA (rRNA) internal transcribed spacer regions showed that a number of strains in the same monophyletic clade as *P. davidi *were able to survive desiccation treatments at high rates whereas others outside that clade were much more sensitive to desiccation [35]. Although this initial study provided important insights into the evolutionary relationships of different *Panagrolaimus *strains and species, it was limited by inclusion of only eleven *Panagrolaimus *strains and the use of only one very distantly related outgroup (*C. elegans*). The evolutionary origins of varying reproductive modes across different *Panagrolaimus *lineages remain unknown, as do the origins of the lineage leading to the Antarctic species *P. davidi*.

Here we provide an extensive evolutionary analysis of thirty-one *Panagrolaimus *species and strains that utilizes both slowly-evolving nuclear loci and a faster-evolving mitochondrial locus to provide insights into the phylogenetic relationships of different lineages and to map the origins of parthenogenetic and hermaphroditic reproductive modes. Strain-specific reproductive modes were analyzed by assaying for the presence of sperm using microscopy techniques. To provide a rough estimate of the general timeline for *P. davidi*'s arrival in Antarctica, we extended a molecular clock approach previously applied to *Caenorhabditis *[36] to approximate the divergence time between *P. davidi *and its closest known relative.

## Results and discussion

### *Panagrolaimus *strains and reproductive mode analysis

We initiated our study by requesting available *Panagrolaimus *strains from the *Caenorhabditis *Genetics Center and other helpful colleagues (see Table 1). Further, in the summer of 2007 we sampled soils from diverse locations around Oregon and discovered seven additional *Panagrolaimus *isolate strains from which laboratory cultures were generated and frozen stocks were made. A total of thirty-one strains were collected from our colleagues and the field that were successfully established in laboratory culture (Table 1). Four additional nematode strains isolated in Monroe, Oregon, USA (LD3, LD6, LD7 and LD8) were used for molecular analyses but were not kept in long-term laboratory culture. We are able to maintain the thirty-one *Panagrolaimus *strains listed in Table 1 at 25°C using standard OP50 *Escherichia coli*-seeded NGM plates. We are also able to cryogenically preserve all strains in glycerol solutions using standard *C. elegans *techniques [1], and to developmentally synchronize lab populations using the hypochlorite treatment method [37]. We discovered that multiple larval and adult developmental stages of *Panagrolaimus *nematodes survived cryogenic storage whereas in *C. elegans *only L1-stage larvae generally survive. We kept track of generation times and brood sizes for two *Panagrolaimus *strains (PS1579 and PS1159 – both parthenogenetic) and found that they experience generation times of approximately seven days and brood sizes of 300–400 eggs under the aforementioned laboratory culturing conditions. Thus, *Panagrolaimus *offers many of the same basic attractive features that have contributed to the outstanding success of the *C. elegans *system. Furthermore, RNA interference-mediated gene silencing has recently been documented to be effective in *P. superbus *and the parthenogenetic *Panagrolaimus *strain PS1162 [38].

**Table 1:** *Panagrolaimus *strains analyzed.

*Strain*	*Other names*	*Geographic origin*	*Source*	*Sperm*	*Reprod.*
AF36	*P. rigidus*	Hungary?	CGC, AB	ND	G
AF40	*P. rigidus *(*Oscheius sp.*)	Hungary?	CGC, AB	ND	G
BSS8	*P.detritophagus*	Iceland	AB	Yes	H
BW287	*P.*sp1 (*C. briggsae*)	Beijing, China	CGC, AB	ND	H
CB1	*P. davidi*	McMurdo Sound region, Antarctica	DW	No	P
DF5050	*P. superbus*	Iceland	AB	ND	G
DL0050	none	Portland, OR, USA	DL	No	P
DL0072	none	Corvallis, OR, USA	DL	No	P
DL0117	none	Portland, OR USA	DL	ND	G
DL0128	none	Salem, OR, USA	DL	ND	G
DL0137	none	Corvallis, OR, USA	DL	No	P
DL0139	none	Corvallis, OR, USA	DL	No	P
DL0180	none	Salem, OR, USA	DL	ND	G
ES1	*P*. "brombeer"	Cologne, Germany	ES	ND	G
ES2	*P*. "brauwciler"	Cologne, Germany	ES	ND	G
ES3	*P*. "mais"	Eifel Mtn, Germany	ES	ND	G
ES5	*P*. "bornheim"	Bonn, Germany	ES	ND	G
ES6	*P*. sp.	Cologne, Germany	ES	ND	G
JB051	DF049	Senegal	JB	No	P
JB115	*P*. sp.	San Bernadino Mtns, CA, USA	JB	No	P
JB131	*Eucephalobus sp.1*	Baja California, Mexico	JB	No	P
JU765	*P*. sp.	Guangxi, China	MAF	Yes	H
PS443	*P*. sp3	Armenia	AB, MAF	ND	G
PS1159	*P*. sp2	N. Carolina, USA	AB, MAF	No	P
PS1162	*P*. sp.	Beijing, China	PS	No	P
PS1579	*P*. sp4	Huntington Gardens, CA, USA	CGC, AB	ND	P
PS1732	*P*. sp5	Sierra Mtns, CA, USA	PS	Yes	H
PS1806	none	Huntington, CA, USA	PS	No	P
PS3966	none	Pasadena, CA, USA	PS	No	P
SN103	none	Susanville, CA, USA	AB	No	P
none	*P.paetzoldi*	Westerscheldt, Netherlands	AB	ND	G

Strains sources are: JB, James G. Baldwin; AB, Ann Burnell; DL, Denver lab; MAF, Marie-Anne Félix; ES, Einhard Schierenberg; PS, Paul W. Sternberg; DW, David Wharton. Modes of reproduction (Reprod.) are: G, gonochoristic; H, hermaphroditic; P parthenogenetic.

We initially distinguished between gonochoristic and self-fertile *Panagrolaimus *strains by carrying out self-fertility tests whereby early larval stage nematodes were individually transferred to plates and then allowed to develop in isolation. Strains repeatedly unable to carry out self-fertilization were defined as gonochoristic – in all cases, mass populations of strains found to be gonochoristic by this criterion were also observed to contain large numbers of mating males. Our definitions of gonochoristic strains were also consistent with previous independent observations [16,23,35].

**Figure 1:**
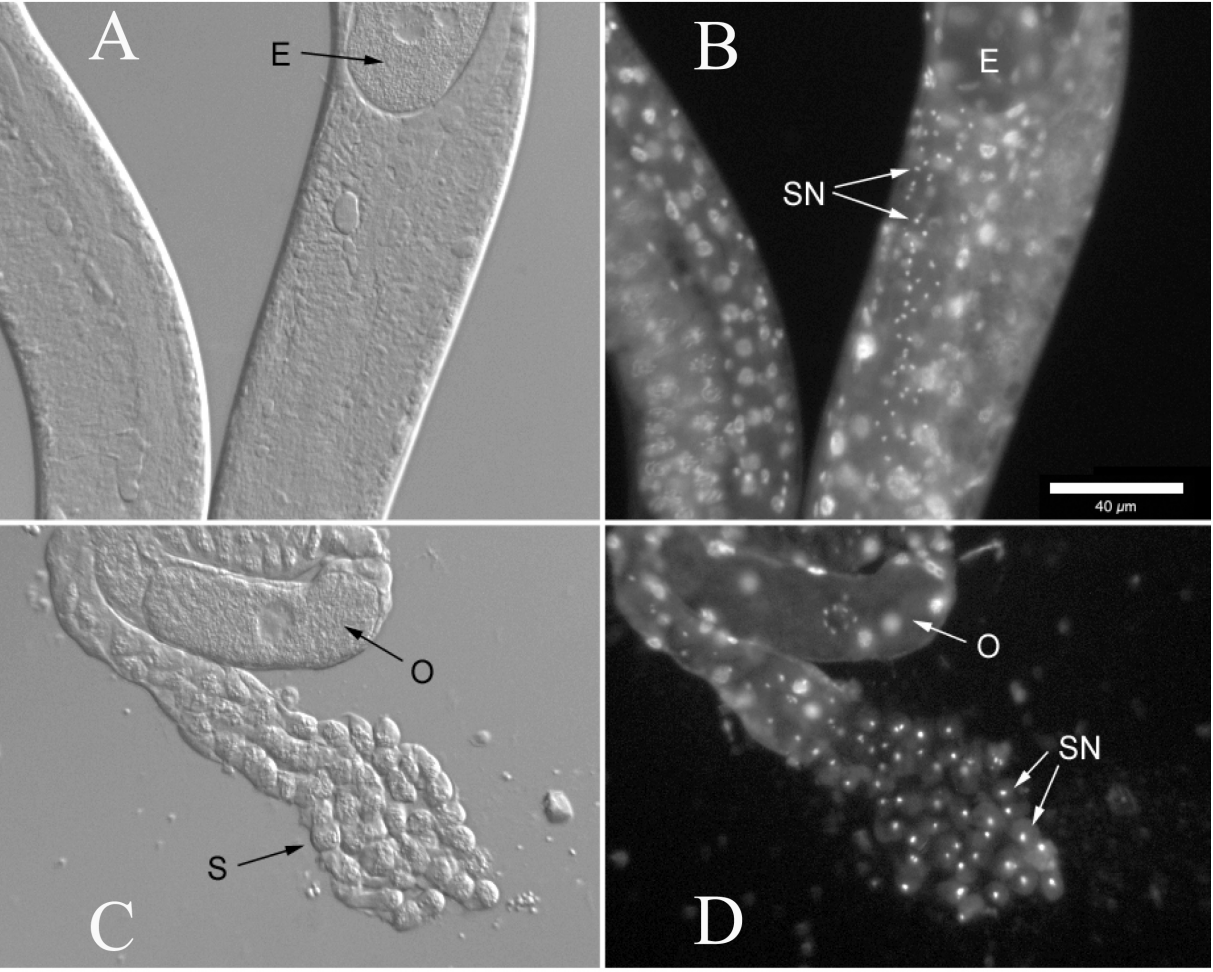
**Identification of sperm and sperm nuclei from hermaphroditic *Panagrolaimus *strain JU765**. DIC imaging was used to identify sperm (left, panels A and C) and epifluorescence imaging was used to identify characteristically compact sperm nuclei (right, panels B and D). See Methods for details. A and B show nematode midbodies; C and D show a dissected spermatheca. Ann egg is indicated by "E", sperm nuclei by "SN", oocytes by "O", and sperm by "S". The scalebar represents 40 μm.

**Figure 2:**
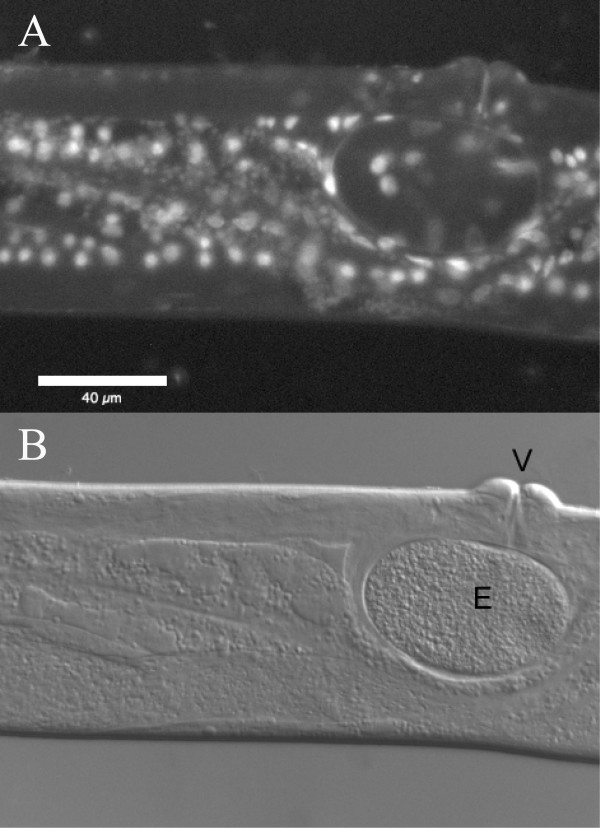
**Lack of sperm and sperm nuclei from parthenogenetic *Panagrolaimus *strain JB115**. DIC imaging was used to search for sperm and epifluorescence to search for compact sperm nuclei (none were observed). See Methods for details. An egg is indicated by "E" and the vulva is indicated by "V". The scalebar represents 40 μm.

For all self-fertile strains, we tested for the presence or absence of sperm using DIC/epifluorescence microscopy techniques (see Methods for details). Three self-fertile *Panagrolaimus *species/strains (JU765, PS1732, *P. detritophagus*) were observed to produce sperm (Figure 1) – we consider these to most likely be hermaphroditic, though we cannot rule out the possibility that the sperm is only used for egg activation and does not contribute genetic material. For all other self-fertile *Panagrolaimus *strains examined, no sperm was detected and we consider them to be parthenogenetic (Figure 2). In mass laboratory populations, males are frequently observed for *P. detritophagus *and occasionally observed for JU765 and PS1732. We have not observed males in any of the parthenogenetic strains, though a focused study on male frequencies has not yet been carried out. Males were observed to be abundant in field populations of the Antarctic species *P. davidi *whereas parthenogenetic females were observed to dominate long-term lab cultures [39].

### *Panagrolaimus *evolutionary relationships

We applied a molecular phylogenetic approach to understand the evolutionary relationships of *Panagrolaimus *species and strains, and to map the origins of reproductive mode transitions in this nematode genus. We PCR-amplified and directly sequenced segments of the 18S and 28S rRNA genes from genomic DNA samples derived from each of the thirty-one *Panagrolaimus *strains (see Methods). Amplified sequences were subjected to multiple alignment using ClustalW in the program MEGA4 [40]; subsequently numerous orthologous outgroup sequences representing diverse nematode taxa of varying evolutionary distances from *Panagrolaimus *were also included in the alignment (sequences obtained from Genbank). After making minor corrections to the alignment based on visual inspection, we performed phylogenetic analyses in MEGA4 using both neighbor-joining (NJ) and maximum parsimony (MP) approaches (see Methods). A total of 436 aligned nucleotide sites were analyzed for 18S rRNA; 623 aligned nucleotide sites were analyzed for 28S rRNA. Concatenated 18S and 28S sequences (1,059 sites total) were used for phylogenetic analyses. Additional analyses involved a segment (778 aligned nucleotide sites) of the more rapidly evolving mitochondrial NADH dehydrogenase subunit 5 (*ND5*) gene to independently test the results of nuclear rRNA analyses and further analyze molecular diversification patterns. *ND5 *sequences were successfully obtained for all *Panagrolaimus *except DL0139 (Corvallis, Oregon, USA) and ES6 (Cologne, Germany). As with the nuclear loci, aligned *ND5 *sequences were subject to MP and NJ phylogenetic analyses in MEGA4 (see Methods).One thousand bootstrap replicates were performed in each analysis to estimate confidence in nodes resulting from the analyses.

**Figure 3:**
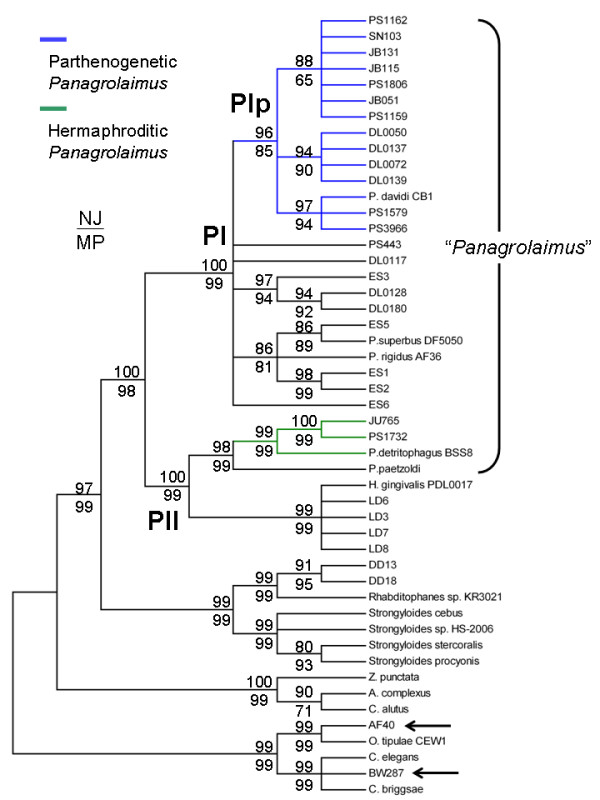
**Nuclear rRNA gene phylogeny for *Panagrolaimus***. Cladogram shown is a 80% bootstrap consensus tree for NJ analysis of aligned 18S and 28S rRNA sequences from *Panagrolaimus *and select outgroup nematode species and strains. The MP phylogeny was highly congruent with the NJ phylogeny shown – see Additional files 1 and 2. Node-specific bootstrap values (1,000 replicates for each analysis) are shown, with NJ values over MP values. All strains and species historically considered as members of the *Panagrolaimus *genus are indicated by the brackets. Arrows denote the positions of two rhabditid strains initially misidentified as *Panagrolaimus*. Blue lines indicate parthenogenetic strains and green lines indicate hermaphroditic strains of *Panagrolaimus*. *H. gingivalis *also reproduces parthenogenetically in infected horses. DD13 and DD18 are two strains isolated from Newport, OR USA that are closely related to *Rhabditophanes sp*. KR3021. All other outgroup sequences were retrieved from Genbank.

NJ and MP analyses of the aligned nuclear rRNA data yielded highly congruent bootstrap consensus trees (Figure 3, Additional files 1, 2). Although there was a single well-supported monophyletic clade (referred to herein as Clade PI) that exclusively contained 25/31 *Panagrolaimus *sequences analyzed, four other *Panagrolaimus *sequences (JU765, PS1732, *P. detritophagus *BSS8, *P. paetzoldi*) were included with a separate monophyletic clade, referred to herein as Clade PII, also containing sequences from *Halicephalobus gingivalis *[41] and related isolates. The sequence from strain AF40 was grouped with the rhabditid *Oscheius tipulae *and the sequence from strain BW287 was grouped with *C. elegans *and its congener *C. briggsae*. A nearly complete mitochondrial genome sequence was previously generated for BW287 which confirmed that it is a strain of *C. briggsae *[4]. These two strains historically identified as members of this genus (AF40, BW287) are clearly members of the very distantly related rhabditid group of nematodes and likely constitute simple accidental cases of misidentification or laboratory contamination.

Our results suggest that a taxonomic revision is required for *Panagrolaimus *since those strains historically identified as members of the genus do not compose a monophyletic group. There are two approaches to solving this dilemma given the phylogeny shown in Figure 3: one would be to remove the four PII *Panagrolaimus *strains from the genus rendering PI the monophyletic *Panagrolaimus *genus; the second would be to reassign *Halicephalobus gingivalis *and related nematodes to the genus *Panagrolaimus *rendering the monophyletic clade containing PI and PII the *Panagrolaimus *genus. Proper systematic revision of this nematode group and its relatives, though clearly necessary, will require a more broad-based analysis involving additional nematode genera (e.g. *Panagrellus*, *Panagrobelus*, *Plectonchus*, *Turbatrix*), more genetic markers and reconsideration of morphological features [42]. Rather than proposing specific taxonomic revisions for the present study whose focus is outside of these specific systematic issues, we will maintain the historical *Panagraimus *identifiers listed in Table 1 and refer to clades PI and PII (Figure 3) to distinguish between different *Panagrolaimus *subgroups.

### Evolution of reproductive mode transitions

We next mapped reproductive modes onto the rRNA consensus phylogeny to identify the evolutionary origins of reproductive mode transitions. Within Clade PI we identified a monophyletic subclade (referred to herein as Clade PIp) that exclusively contained all of the parthenogenetic *Pangrolaimus *strains and species (Figure 3). This observation indicates a single evolutionary origin of parthenogenetic reproduction in *Panagrolaimus*.

The remaining Clade PI species and strains outside of Subclade PIp were all gonochoristic – Additional file 3 provides a summary of knowledge on species boundaries among the PI gonochoristic strains.

The three hermaphroditic *Panagrolaimus *species and strains (*P. detritophagus*, JU765, PS1732) were all placed outside of Clade PI and were included in a separate monophyletic clade (PII) containing one gonochoristic *Panagrolaimus *species (*P. paetzoldi*) along with the opportunistic equine (and sometimes human) pathogen *Halicephalobus gingivalis *[41] and related nematodes (LD3, LD6, LD7, LD8) that we did not maintain in long-term culture. The nuclear phylogeny suggests a single origin of hermaphroditism in PII, although the small number of PII *Panagrolaimus *strains available for analysis precludes any strong conclusions.

In the PI clade there appears to have been a single evolutionary transition to parthenogenesis; among the PII *Panagrolaimus *strains the phylogeny suggests a single transition to hermaphroditism. By contrast, there is evidence for at least five independent transitions to hermaphroditism in *Pristionchus *[20], three in *Oscheius *[17], and three in *Caenorhabditis *(K. Kiontke, pers. comm.). Although this might superficially suggest a reduced plasticity in sex determination mechanisms regulating hermaphroditic versus female development in *Panagrolaimus *versus other well-studied nematode genera, *Panagrolaimus *likely suffers more than the other genera in terms of taxon undersampling – our observations in PII are based on only four *Panagrolaimus *strains. Although a more rigorous sampling of nematodes in the PII clade is required for a robust understanding of reproductive mode transitions in this group, it is interesting to note that *H. gingivalis *in PII reproduces parthenogenetically in infected horses [41]. PII thus might represent a nematode group of exceptional sex determination plasticity able to explore multiple reproductive mode strategies.

### Molecular diversification in the parthenogens

In addition to illuminating the broad-based evolutionary relationships of all nematodes historically placed in the *Panagrolaimus *genus, our rRNA analyses also provided evidence for subdivision of the PIp clade of parthenogens into three subclades (Figure 3). To further investigate divergence in the PIp clade, we analyzed a segment of the more rapidly evolving *ND5 *gene from a subset of *Panagrolaimus *strains to independently test the results of nuclear rRNA analyses and further analyze diversification within the PIp clade of parthenogens. Sequences were successfully obtained for 13/14 of the PIp parthenogens – we were repeatedly unable to PCR amplify the *ND5 *fragment from DL0139 (Corvallis, Oregon, USA).

**Figure 4:**
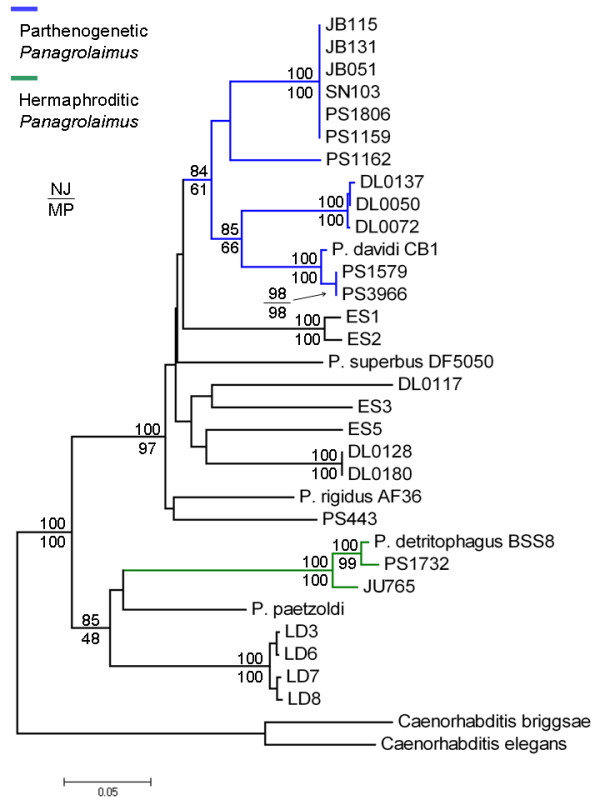
**Mitochondrial *ND5 *gene phylogeny for *Panagrolaimus***. Phylogram shows relationships among a subset of *Panagrolaimus ND5 *sequences (those able to be successfully amplified) inferred from NJ phylogenetic analysis (see Methods). Scale bar shows 0.05 substitutions per site. Bootstrap values resulting from NJ and MP analyses are shown for select nodes (e.g. those supporting PI, PIp and HI). Additional files 4 and 5 show complete phylograms with all bootstrap values for NJ and MP analyses, respectively. Blue lines indicate parthenogenetic strains and green lines indicate hermaphroditic strains.

The mitochondrial *ND5 *gene NJ phylogeny (Figure 4, Additional file 4) was highly congruent with the nuclear rRNA phylogeny (Figure 3). The PI, HI and PIp clades were each supported with strong (> 80%) boostrap support in NJ analysis of mtDNA sequence. Clade PIp was also monophyletic in MP analysis of mtDNA, though with low boostrap support; similarly Clade PII was monophyletic in MP mtDNA analysis with low bootstrap support (see Figure 4). Clade PI, however, was strongly supported in both NJ and MP analyses of the *ND5 *sequences; furthermore, topological arrangement of all strain analyzed was identical for NJ and MP analyses of *ND5 *(Additional files 4, 5). The distribution of parthenogenetic strains in Clade PIp subclades in *ND5 *phylogenetic analyses was the same for that of nuclear rRNA analyses. Among the thirteen PIp *Panagrolaimus *parthenogenetic strains considered in mtDNA analysis, seven unique *ND5 *haplotypes were observed. One haplotype was shared by six strains – three were from California, USA, the fourth was from North Carolina, USA, the fifth was from Baja California, Mexico and the sixth was from Senegal. The sharing of a mtDNA haplotype between strains from North America with one from the west coast of Africa might indicate that *Panagrolaimus *strains are able to very rapidly disperse across large geographic distances, as has been proposed for *C. elegans *where identical mitochondrial genome haplotypes have also been observed for geographically disparate isolates [43]. Like *Caenorhabditis *and *Pristionchus *[47,48], *Panagrolaimus *has recently been described to associate with arthropod hosts that might aid in its geographic dispersal [49]. The three PIp Oregon strains analyzed formed a well-supported monophyletic group and all had highly similar *ND5 *haplotypes. Strain PS1162 from China had a unique *ND5 *haplotype. PS1579 and PS3966 were found to share a haplotype. *P. davidi *CB1 and these two California strains (PS1579, PS3966) were again found to form a well-supported monophyletic clade and share highly similar *ND5 *sequences (Figure 4), suggesting that the Antarctic strain *P. davidi *CB1 and these strains from California shared a common ancestor in the not-too-distant past.

### Arrival and evolution of *P. davidi *in Antarctica

Two independent molecular phylogenies presented here (Figure 3, Figure 4) show very close evolutionary relationships between two PIp parthenogens from southern California (PS1579, PS3966) and *P. davidi *CB1 from Antarctica. To extend upon the phylogenetic results, we applied a molecular clock approach to estimate the number of nematode generations that separate PS1579 and *P. davidi *CB1. Under the neutral theory of molecular evolution [44], the rate of mutation is expected to be equal to the rate of substitution at neutral sites, and the time to the most recent common ancestor (TMRCA) for any two taxa for which there is relevant DNA sequence data can be estimated by the equation T = *K*/(2 *μ*) where T is the time to the most recent common ancestor, *K *is a measure of the neutral molecular divergence between the compared taxa and *μ *is the mutation rate [45]. For the nematode *C. elegans*, there are direct estimates of the per-generation nuclear and mtDNA mutation rates based on mutation-accumulation line systems [5,6]. The nuclear rate estimate was recently employed in an analysis of divergence times among *Caenorhabditis *species utilizing an internally calibrated molecular clock approach [36]. We applied a similar (though externally calibrated) approach here, assuming that the *Panagrolaimus *mtDNA *μ *is the same as that estimated for *C. elegans*, to estimate the TMRCA for PS1579 from California and *P. davidi *from Antarctica.

To provide a broader mtDNA data set for evolutionary rate analysis, we collected additional mtDNA protein-coding gene sequences for PS1579 and *P. davidi *CB1; data examined includes sequences from Cytochrome b, Cytochrome Oxidase I (*COI*), *ND1 *and *ND5*; 2,247 bp, 749 codons total. We employed DnaSP v4.1 [46] to calculate the rate of silent-site substitution (*d*_*S*_), a value often used to estimate neutral evolutionary rates, in the concatenated protein-coding gene sequence pairwise alignment – a *d*_*S *_value of 0.027 was estimated from the PS1579-*P. davidi *alignment. Employing the mtDNA *d*_*S *_(0.027) estimate suggests a rough TMRCA value of 140,206 nematode generations for PS1579 and *P. davidi*. Translating the number of nematode generations into actual (chronological) time is problematic due to uncertainties in the numbers of generations experienced by *Panagrolaimus *in the wild – a general problem for nematodes [36]. Although PS1579 was observed to experience generation times of seven days in the lab at 25°C under food-rich conditions, suggesting approximately fifty-two generations per year, massive environmental differences between the lab and nature (e.g. temperature, food supply type and abundance) almost certainly affect the number of *Panagrolaimus *generations per year in nature. In particular, *P. davidi*'s native Antarctic environment likely provides conditions where nematodes are able to reproduce for a few months (if not weeks) per year [39]. By contrast, the much warmer climate of southern California experienced by PS1579 likely enables a much greater number of generations per year. Additional file 6 shows the relationship between TMRCA estimates (in years) for PS1579 and *P. davidi *and assumed numbers of nematode generations per year. If PS1579 and *P. davidi *have both experienced as few as ten generations per year since their divergence, our analyses based on *d*_*S *_roughly approximate a TMRCA of only ~14,000 years. Even if these two taxa have each experienced only one generation per year since their divergence, a TMRCA of only ~140,000 years is approximated. These TMRCA measures should be interpreted with great caution, however, given the untested assumptions underlying these particular molecular clock analyses – for example, that mtDNA mutation rates are the same for *C. elegans *and *Panagrolaimus *and that the rate of mutation equals the rate of silent-site substitution. Nonetheless, the very close genetic distances between PS1579, PS3966 and *P. davidi *CB1 along with the phylogenies presented in Figures 3 and 4 also generally suggest that *P. davidi *colonized Antarctica in the very recent past.

Did *P. davidi *adaptively evolve extreme cold tolerance after its arrival in Antarctica? Although a current comparative cryotolerance study involving *P. davidi *showed that this species was unique in its extreme cold tolerance as compared to other nematode species examined, the other nematodes used for comparison in the study were very distantly related to *P. davidi *– the closest relative was *P. rigidus *that our phylogenetic and genetic distance analyses show is very distantly related to *P. davidi*. Future comparative cryotolerance studies involving *P. davidi*'s closely-related parthenogen relatives (e.g. PS1579) would shed light on whether extreme cold tolerance is a feature unique to *P. davidi*, or if it is shared by its closely-related parthenogenetic relatives. If the latter scenario is true, it would suggest that *P. davidi *arrived in Antarctica in a "pre-adapted", cryotolerant state. If the latter scenario is not true, it would suggest that *P. davidi *was able to very rapidly adapt to the harsh Antarctic environment.

## Conclusion

Our study sheds light on the evolutionary relationships of *Panagrolaimus *nematodes, showing that the traditional genus is paraphyletic. Our analyses suggest a single origin of parthenogenesis in *Panagrolaimus *and provide evidence that *P. davidi *arrived in Antarctica in the very recent evolutionary past. This nematode genus offers an outstanding model for analyzing the evolutionary and biological causes of transitions from gonochoristic to parthenogenetic reproduction, and the adaptive evolution of extreme cold tolerance. We also note here that a brief soil survey effort in northwest Oregon performed by a single undergraduate student (C. F. H.) resulted in the discovery two probable new gonochoristic species of *Panagrolaimus *(DL0117 and DL0128/DL0180 – see Additional file 3) and a new major clade of PIp parthenogens. The worldwide diversity of *Panagrolaimus *and other nematode species in the soil and other ecologies likely remains vastly undersurveyed, being a key limiting factor in our understanding of evolutionary processes in this diverse animal phylum.

## Methods

### Strain sources and maintenance

Thirty-one strains of *Panagrolaimus *were obtained from various sources for analysis (Table 1). All nematodes were cultured on standard NGM agarose plates, seeded with the OP50 strain of *E. coli *as a food source. Nematodes were maintained at 25°C in Percival incubators, and transferred to fresh plates once a week. Frozen stocks were created for each *Panagrolaimus *strain following standard *C. elegans *freezing protocols [1]. *C. elegans *standard bleaching protocols for developmental synchronization [37] were also applied to the *Panagrolaimus *strains.

### Microscopy methods for sperm identification

At least 10 gravid worms from each strain were placed into a droplet of ethanol on a microscope slide, and the ethanol was allowed to evaporate completely. A 10 μl drop of PBS containing 20 μg/ml of DAPI (4',6-diamidino-2-phenylindole) was added to the ethanol-fixed worms to label the nuclei [50]. A coverslip was placed over the labeled worms, and they were observed under epifluorescence. Sperm were identified by their characteristic compact nuclei. When sperm nuclei were detected for a particular worm species, the identification was verified by dissecting additional worms from that species in Sperm Medium (SM1: 50 mM HEPES pH7.0, 50 mM NaCl, 25 mM KCl, 1 mM MgSO_4_, 5 mM CaCl_2_, 20 mM Glucose pH7.0) containing DAPI (20 μg/ml). The sperm were identified under differential interference contrast (DIC) optics, and their nuclei were observed under epifluorescence.

### PCR amplification and DNA sequencing

Nematode populations were allowed to expand until food sources were exhausted, after which the worms were harvested for DNA extraction, using previously-established protocols for *C. elegans *[43]. For nuclear loci, standard polymerase chain reaction (PCR) amplifications for 18S and 28S rRNA gene sequences were performed in 50 μL reactions as previously described [43]. The 18S rRNA fragment was amplified using primers 18A and 26B [51] and the 28S rRNA fragment was amplified using primers #537F and #531R [42]. The mitochondrial segment analyzed across multiple *Panagrolaimus *strains (*ND5 *gene) was amplified using the Roche Expand Long Range PCR kit with primers 39F and 58R that are nearly universal nematode mtDNA PCR primers [52]. Additional mtDNA segments exclusively analyzed in PS1579 and *P. davidi *CB1 resulted from two additional PCR amplifications: COIF to 40R and PS5F to 36R. Universal nematode mtDNA primers were again employed, with the exception of PS5F that we designed specific to PS1579 mtDNA sequences. All primer sequences are available upon request. PCR products were purified using solid phase reversible immobilization techniques [53] and then used as template for cycle sequencing. PCR primers were used for sequencing, along with internal primers when necessary. Cycle sequencing was carried out by 25 cycles of denaturation at 96°C for 30 seconds, annealing at 50°C for 15 seconds, and extension at 60°C for 4 minutes. Unincorporated dye terminators were removed by ethanol precipitation and DNA sequencing reactions were analyzed using an ABI 3730 capillary sequencer at the OSU Center for Genome Research and Biocomputing. New DNA sequences used in this study were deposited in Genbank under accession numbers FJ590596-FJ591048.

### Alignment and evolutionary analyses

Alignment and phylogenetic analysis procedures were carried out identically for nuclear and mtDNA sequences. DNA sequence alignments and phylogenetic analyses were performed using MEGA4 [40]. Alignments were done using the ClustalW feature offered by MEGA4 – gap opening penalties were set at 15 and gap extension penalties at 6.66. The IUB DNA weight matrix was utilized. After alignment, the ends of sequences were trimmed off to create aligned sequences of equal character length and then the 18S and 28S rDNA sequences were joined for subsequent phylogenetic analyses. The *ND5 *mtDNA sequences were analyzed independently from the nuclear sequences. After automatic alignment, minor manual adjustments to the alignment were made prior to phylogenetic analysis. MEGA4 was also applied for MP and NJ phylogenetic analyses. The maximum-composite likelihood molecular evolution model was used for neighbor-joining analyses. 1,000 bootstrap replicates were performed for all phylogenetic testing. We estimated *d*_*S *_values from PS1579-*P. davidi *CB1 mtDNA alignment files using DNAsp v4.1 [46].

## Abbreviations

*d*_*S*_: rate of silent-site substitution; mtDNA: mitochondrial DNA; MP: maximum parsimony; NJ: neighbor joining; rRNA: ribosomal RNA; TMRCA: time to the most recent common ancestor

## Authors' contributions

SCL performed all mitochondrial sequencing experiments and evolutionary analyses, and contributed to manuscript writing; LAD contributed to nuclear sequencing experiments and evolutionary analyses, reproductive mode analyses and manuscript writing; CH contributed to nuclear sequencing experiments, set up the mating tests and discovered the Oregon *Panagrolaimus *strains; SW contributed to strain maintenance and reproductive mode analyses; W-SL contributed to microscopy experiments; CWL contributed to microscopy experiments; DRD conceived the study and contributed to evolutionary analyses and manuscript writing. All authors read and approved the manuscript.

## Supplementary Material

Additional file 1 **NJ phylogram for nuclear rRNA sequences.** Complete bootstrap consensus phylogram for NJ analysis, including bootrap support across all nodes, is shown. Scale bar shows 0.01 substitutions per site. Click here for file

Additional file 2 **MP phylogram for nuclear rRNA sequences**. Complete bootstrap consensus phylogram for MP analysis, including bootrap support across all nodes, is shown. Scale bar shows 50 substitutions. Click here for file

Additional file 3 **Species boundaries among the PI gonochoristic strains**. The note provides a brief description of knowledge on species boundaries among gonochoristic *Panagrolaimus *the PI clade, and a table of nuclear and mitochondrial genetic distances between these strains. Click here for file

Additional file 4 **NJ phylogram for mitochondrial *ND5 *sequences**. Complete bootstrap consensus phylogram for NJ analysis, including bootrap support across all nodes, is shown. Scale bar shows 0.05 substitutions per site. Click here for file

Additional file 5 MP phylogram for mitochondrial *ND5 *sequences. Complete bootstrap consensus phylogram for MP analysis, including bootrap support across all nodes, is shown. Scale bar shows 20 substitutions. Click here for file

Additional file 6 TMRCA estimates for PS1579 and *P. davidi *CB1. TMRCA estimate (in years) is shown on the y axis assumed number of nematode generations per year is shown on the x axis. Relationships are shown on a log-log plot. See Methods and main text for details. Click here for file
