# Supplementary figures and images for: Molecular evolution in Panagrolaimus nematodes: origins of parthenogenesis, hermaphroditism and the Antarctic species P. davidi
Source: BMC Evol Biol. 2009 Jan 16;9:15. doi: 10.1186/1471-2148-9-15 (PMC2632994; doi:10.1186/1471-2148-9-15)

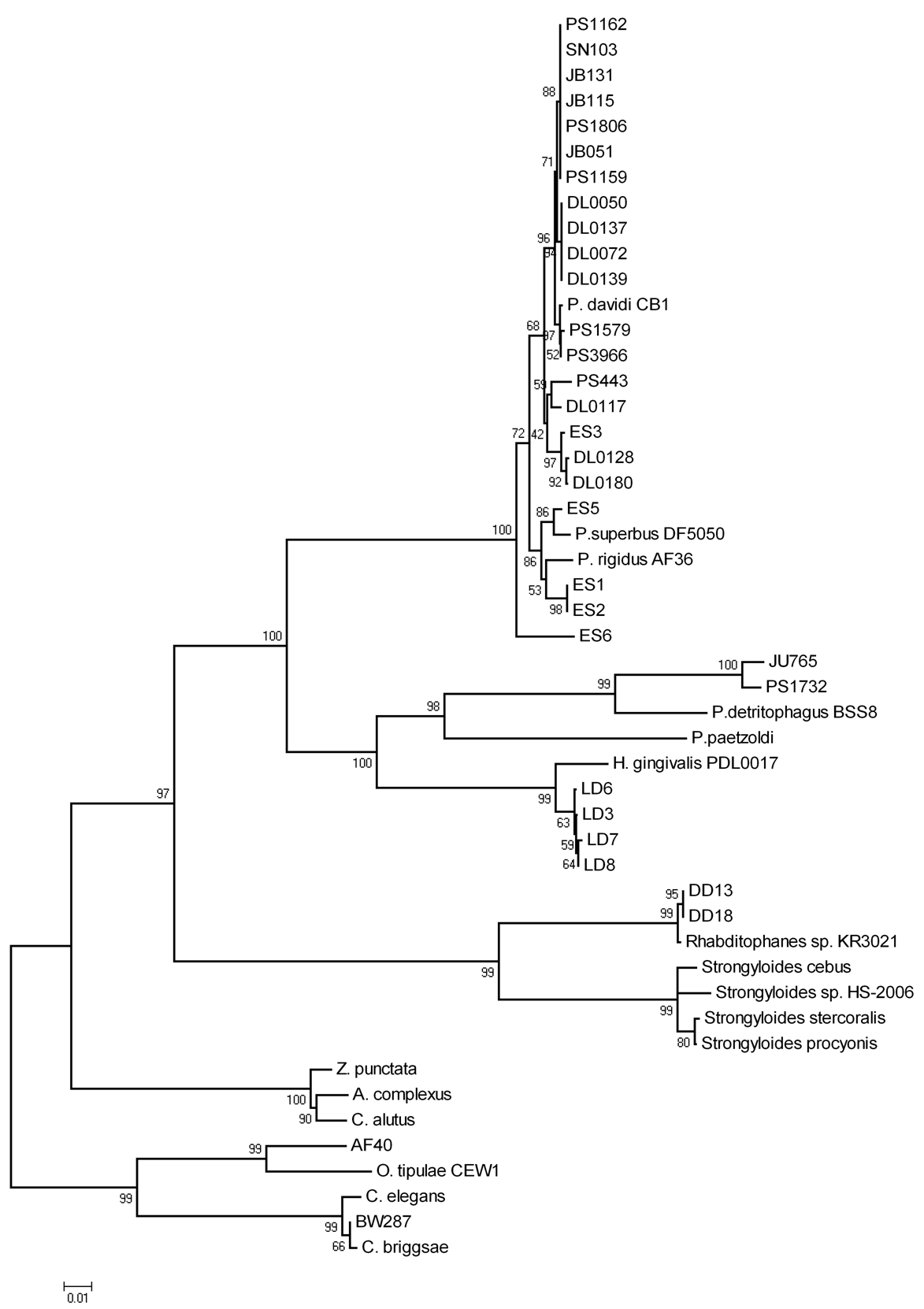

Supplement: Additional file 1 — NJ phylogram for nuclear rRNA sequences. Complete bootstrap consensus phylogram for NJ analysis, including bootrap support across all nodes, is shown. Scale bar shows 0.01 substitutions per site. [file 1471-2148-9-15-S1.tiff]

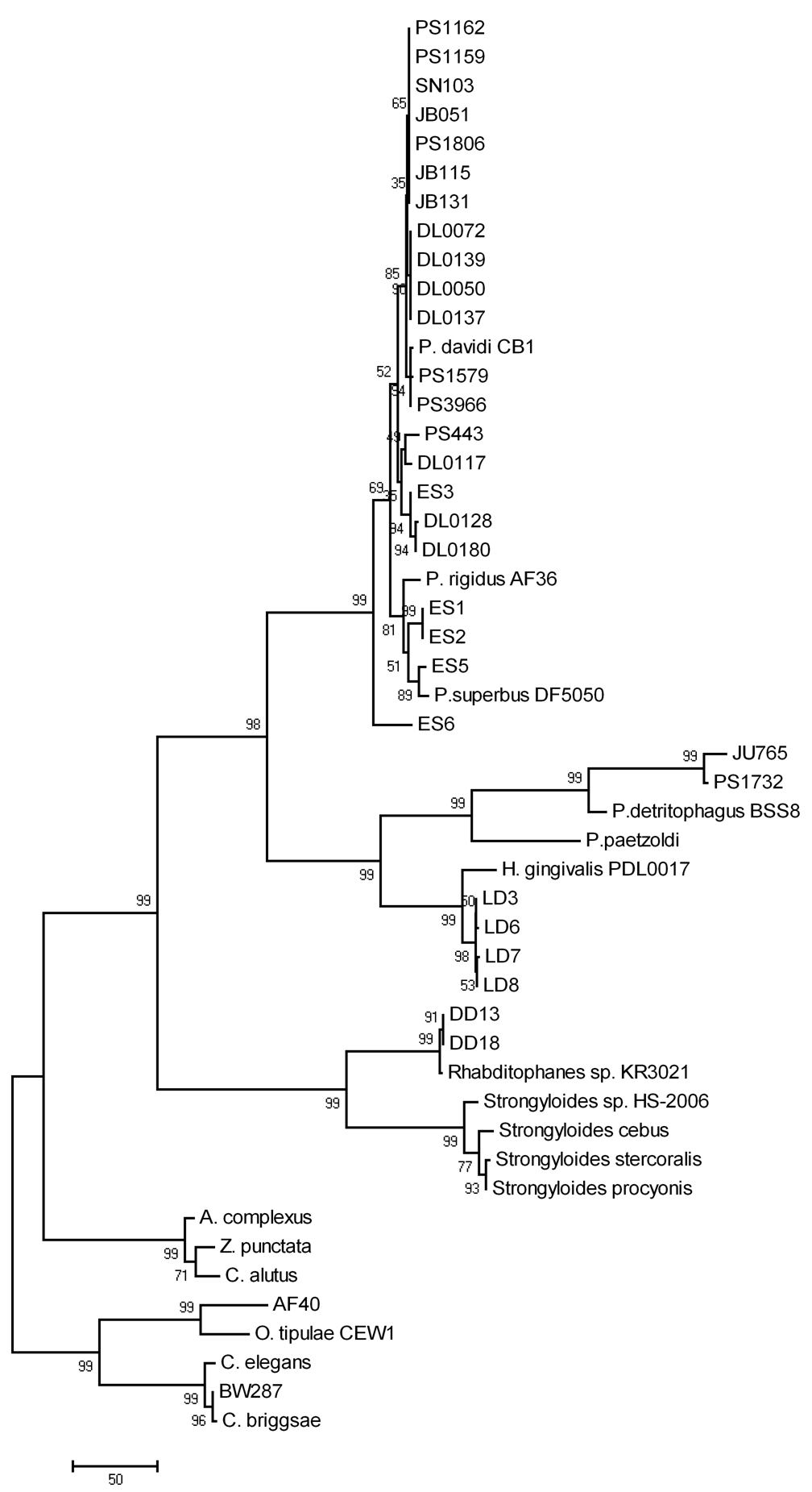

Supplement: Additional file 2 — MP phylogram for nuclear rRNA sequences. Complete bootstrap consensus phylogram for MP analysis, including bootrap support across all nodes, is shown. Scale bar shows 50 substitutions. [file 1471-2148-9-15-S2.tiff]

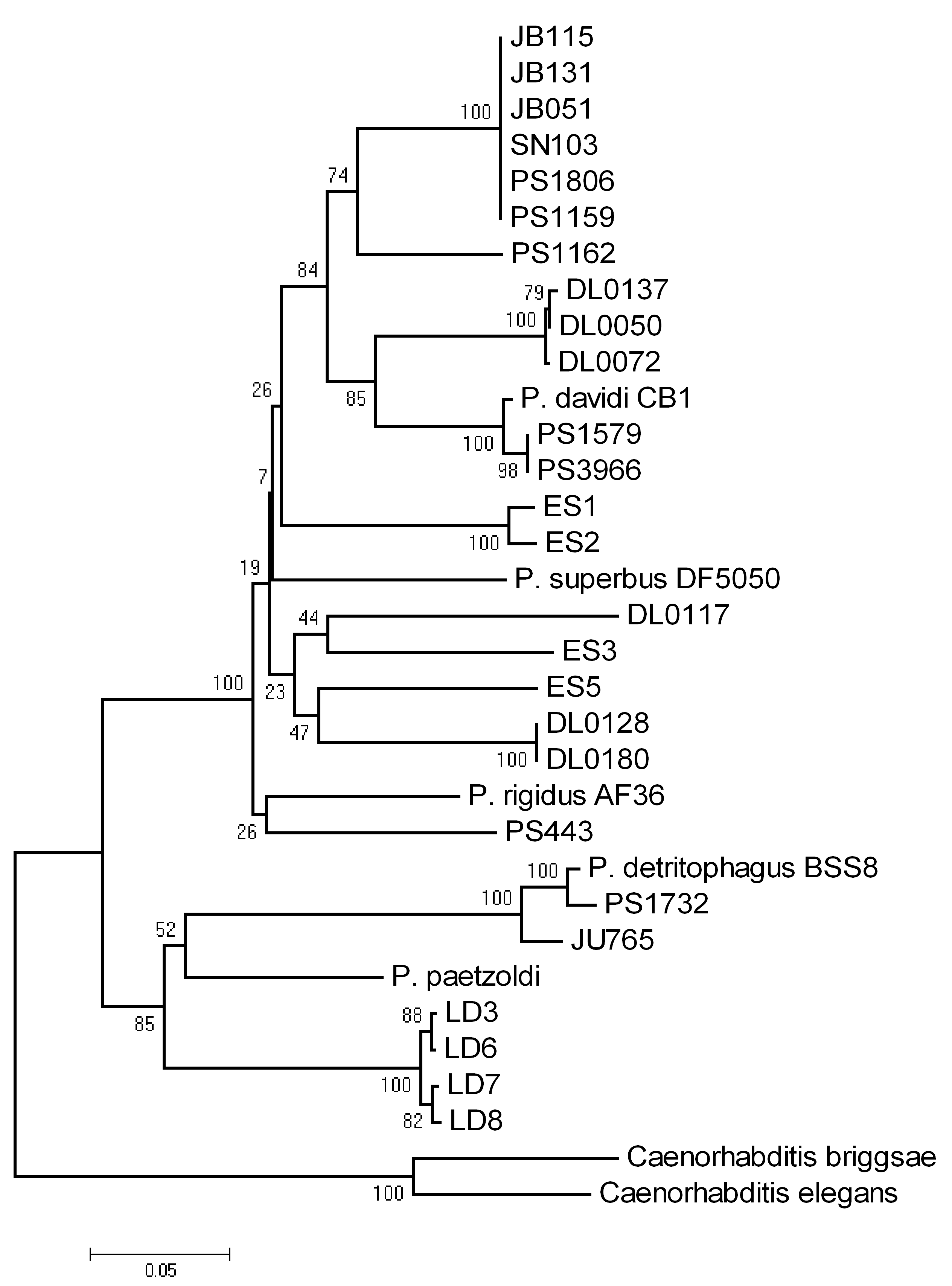

Supplement: Additional file 4 — NJ phylogram for mitochondrial ND5 sequences. Complete bootstrap consensus phylogram for NJ analysis, including bootrap support across all nodes, is shown. Scale bar shows 0.05 substitutions per site. [file 1471-2148-9-15-S4.tiff]

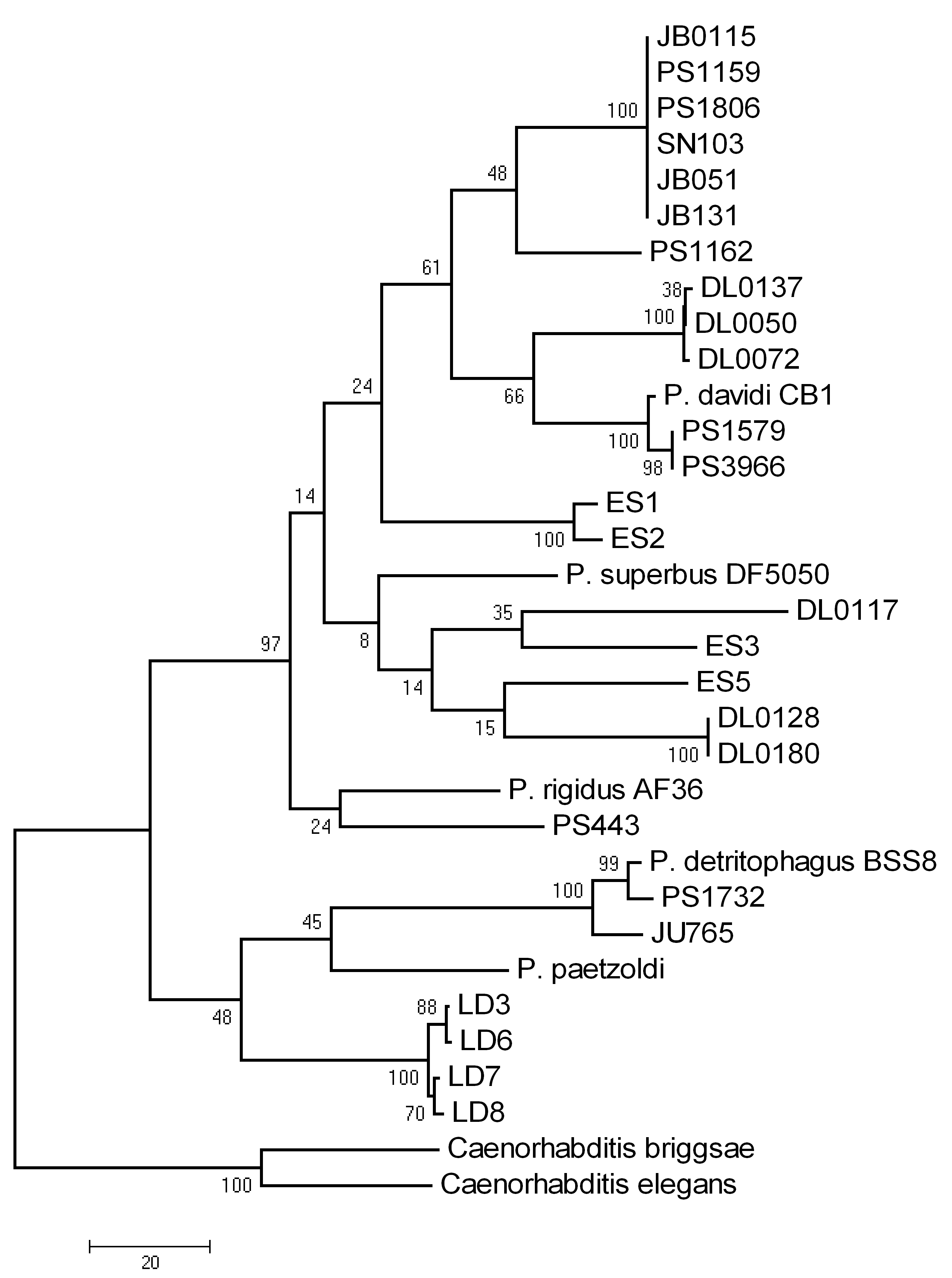

Supplement: Additional file 5 — MP phylogram for mitochondrial ND5 sequences. Complete bootstrap consensus phylogram for MP analysis, including bootrap support across all nodes, is shown. Scale bar shows 20 substitutions. [file 1471-2148-9-15-S5.tiff]

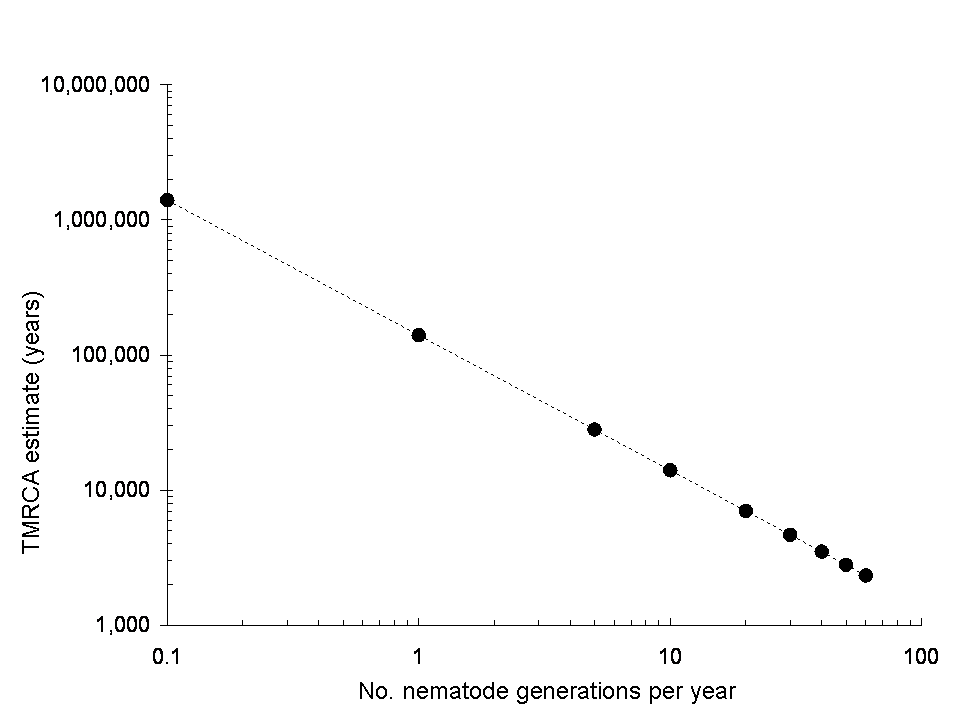

Supplement: Additional file 6 — TMRCA estimates for PS1579 and P. davidi CB1. TMRCA estimate (in years) is shown on the y axis assumed number of nematode generations per year is shown on the x axis. Relationships are shown on a log-log plot. See Methods and main text for details. [file 1471-2148-9-15-S6.tiff]
