# Supplementary material for: Molecular evolution in Panagrolaimus nematodes: origins of parthenogenesis, hermaphroditism and the Antarctic species P. davidi
Source: BMC Evol Biol. 2009 Jan 16;9:15. doi: 10.1186/1471-2148-9-15 (PMC2632994; doi:10.1186/1471-2148-9-15)
Supplement: Additional file 3 — Species boundaries among the PI gonochoristic strains. The note provides a brief description of knowledge on species boundaries among gonochoristic Panagrolaimus the PI clade, and a table of nuclear and mitochondrial genetic distances between these strains. [file 1471-2148-9-15-S3.doc]

**Species boundaries among the PI gonochoristic strains**

Among the PI gonochoristic species and strains, ES1 and ES2 from Cologne, Germany produce viable progeny in the lab (E. Schierenberg, pers. comm.) and crosses done by us show that DL0128 and DL0180 from Salem, Oregon, USA produce viable progeny. For each of these two pairs, genetic distances at nuclear and mitochondrial sequences analyzed are very small, as expected (see table of genetic distances at the end of this note). ES1 and ES2 had identical nuclear rRNA gene sequences and 2% divergence at the mitochondrial locus; DL0128 and DL0180 showed 0.5% sequence divergence at the nuclear loci and had identical mitochondrial sequences. Aside from these two cases, genetic distances for all other possible pairwise comparisons were greater than 1% for the nuclear loci and greater than 16% for the mitochondrial locus. *P. rigidus* AF36 and PS443 are reported to not produce progeny upon setting up crosses [16]. We performed two additional sets of crosses involving newly discovered gonochoristic strains from Oregon: DL0128 X ES3 (nuclear divergence = 1.5%, mitochondrial divergence = 19%) and DL0117 X ES3 (nuclear divergence = 1.9%, mitochondrial divergence = 19%). Neither of these attempted crosses resulted in progeny. All other possible PI gonochoristic strain pairs (other than ES1 & ES2 and DL0128 and DL0180) display greater pairwise nuclear sequence divergence and equally high mitochondrial divergence, the latter almost certainly being saturated with changes for most comparisons [43]. Given these results, we suggest that there are most likely nine distinct biological species among the eleven PI gonochoristic strains shown in Figure 3, with ES1 and ES2 constituting a common species, DL0128 and DL0180 being the same species, and the remaining seven gonochoristic PI strains each constituting single representatives from different unique species. Systematic mating tests, to be performed for a future study after additional environmental sampling is carried out, will be required to precisely define these biological species boundaries.

| **Nuclear distances** | |  |  |  |  |  |  |  |  |  |  |
| --- | --- | --- | --- | --- | --- | --- | --- | --- | --- | --- | --- |
|  | ES1 | ES6 | ES2 | ES3 | ES5 | DF5050 | AF36 | PS443 | DL0117 | DL0128 | DL0180 |
| ES1 |  |  |  |  |  |  |  |  |  |  |  |
| ES6 | 0.092 |  |  |  |  |  |  |  |  |  |  |
| ES2 | 0 | 0.092 |  |  |  |  |  |  |  |  |  |
| ES3 | 0.05 | 0.074 | 0.05 |  |  |  |  |  |  |  |  |
| ES5 | 0.034 | 0.091 | 0.034 | 0.058 |  |  |  |  |  |  |  |
| DF5050 | 0.033 | 0.087 | 0.033 | 0.053 | 0.018 |  |  |  |  |  |  |
| AF36 | 0.036 | 0.092 | 0.036 | 0.048 | 0.045 | 0.046 |  |  |  |  |  |
| PS443 | 0.06 | 0.073 | 0.06 | 0.027 | 0.064 | 0.062 | 0.054 |  |  |  |  |
| DL0117 | 0.056 | 0.065 | 0.056 | 0.019 | 0.062 | 0.055 | 0.051 | 0.022 |  |  |  |
| DL0128 | 0.05 | 0.077 | 0.05 | 0.015 | 0.058 | 0.053 | 0.049 | 0.038 | 0.029 |  |  |
| DL0180 | 0.05 | 0.074 | 0.05 | 0.009 | 0.058 | 0.053 | 0.049 | 0.036 | 0.026 | 0.005 |  |
| **Mitochondrial distances** | | |  |  |  |  |  |  |  |  |  |
|  | ES1 | ES2 | ES3 | ES5 | DF5050 | AF36 | PS443 | DL0117 | DL0128 | DL0180 |  |
| ES1 |  |  |  |  |  |  |  |  |  |  |  |
| ES2 | 0.02 |  |  |  |  |  |  |  |  |  |  |
| ES3 | 0.21 | 0.21 |  |  |  |  |  |  |  |  |  |
| ES5 | 0.19 | 0.19 | 0.17 |  |  |  |  |  |  |  |  |
| DF5050 | 0.19 | 0.19 | 0.21 | 0.18 |  |  |  |  |  |  |  |
| AF36 | 0.19 | 0.19 | 0.19 | 0.19 | 0.16 |  |  |  |  |  |  |
| PS443 | 0.20 | 0.21 | 0.17 | 0.18 | 0.18 | 0.16 |  |  |  |  |  |
| DL0117 | 0.24 | 0.24 | 0.19 | 0.21 | 0.22 | 0.22 | 0.22 |  |  |  |  |
| DL0128 | 0.18 | 0.19 | 0.19 | 0.16 | 0.18 | 0.18 | 0.19 | 0.21 |  |  |  |
| DL0180 | 0.18 | 0.19 | 0.19 | 0.16 | 0.18 | 0.18 | 0.19 | 0.21 | 0.00 |  |  |
